# Self-perceived quality of life, health, and physical activity among older adults: the roles of marital status and residence during the COVID-19 pandemic

**DOI:** 10.3325/cmj.2025.66.390

**Published:** 2025-12

**Authors:** Nada Pjevač Keleminić, Venija Cerovečki, Mirjana Kujundžić Tiljak

**Affiliations:** 1 Department of Family Medicine, Andrija Štampar School of Public Health, University of Zagreb, School of Medicine, Zagreb, Croatia; 2 Health Center Zagreb – Centar, Zagreb, Croatia; 3 Department of Medical Statistics, Epidemiology, and Medical Informatics, Andrija Štampar School of Public Health, University of Zagreb School of Medicine, Zagreb, Croatia; Pjevač Keleminić et al: Self-perceived quality of life, health, and physical activity among older adults

## Abstract

**Aim:**

To examine the associations between marital status, place of residence, self-reported health status, quality of life, and physical activity among older adults during the COVID-19 pandemic.

**Methods:**

This cross-sectional study enrolled 962 participants aged 65 and older, surveyed between March 2020 and May 2023. Respondents were categorized according to marital status (married/living with a partner, single, divorced, widowed) and place of residence (own home vs nursing home). Standardized instruments were used: the Short Form Health Survey-36 for health status, the Personal Well-being Index for quality of life, and the Croatian short version of the International Physical Activity Questionnaire.

**Results:**

Respondents who were married or living with a partner reported significantly higher levels of physical activity, better physical and mental health, and greater life satisfaction than single, divorced, or widowed respondents (*P* < 0.001). Community-dwelling respondents scored significantly higher on most health and quality-of-life indicators than nursing home residents, except for perceived future security.

**Conclusion:**

Marital status and living arrangements significantly affected the self-perceived health, physical activity, and quality of life of older adults during the COVID-19 pandemic. The results emphasize the importance of social support and residential context in promoting healthy aging.

Population aging represents one of the most important demographic trends in contemporary societies. Various classifications of older adults have been proposed, but the most widely accepted categorization divides them into three groups: young-old (65-74 years), middle-old (75-84 years), and old-old (85 years and older) (1). According to the World Health Organization (WHO), individuals aged 60 to 75 years are considered older adults, those aged 76 to 90 are classified as elderly, and those over 90 years are considered very old (2). Over the past 160 years, average life expectancy has increased by approximately 40 years and continues to rise by about three months annually. This increase, combined with declining birth rates, has caused profound demographic shifts, particularly in high-income countries, including Croatia (3). According to the 2021 Census, individuals aged 65 years and older comprise 22.3% of Croatia’s total population. Within this senior demographic, there is a preponderance of women (58.4%) (4). Data from the Croatian Bureau of Statistics and Eurostat further confirm the continuing trend of population aging in Croatia (5). This demographic shift is exacerbated by the emigration of younger individuals and entire families, a process that has intensified since Croatia joined the European Union (6).

The quality of life in older adults is shaped by an interplay of health status, social support, economic security, and environmental factors. Although socio-economic and political circumstances influence subjective assessments of well-being, improved material conditions do not necessarily translate into greater life satisfaction (7-9). While enhancing poor living conditions can initially improve subjective well-being, the effect plateaus beyond a certain threshold (10).

The WHO defines quality of life holistically as an individual’s subjective perception of their place in life, shaped by the cultural, social, and environmental contexts in which they live (11,12). The International Well-being Group emphasizes the multidimensionality of quality of life, which encompasses domains such as health, personal relationships, safety, community conectedness, and perceived future security (13,14). Self-rated health is strongly associated with overall quality of life in older adults (15,16).

Physical and mental health commonly decline with age, and self-perceived health is a key indicator of functional status and well-being. Older adults who perceive their health as good typically report a significantly higher quality of life than those who rate their health poorly (17). Regular physical activity has been associated with improved quality of life and increased longevity, although findings vary across populations and study designs (18,19). The Centers for Disease Control and Prevention recognize physical activity as one of the most effective strategies for promoting health and preventing chronic diseases (20).

Older adults were particularly vulnerable to the COVID-19 pandemic due to age-related immunosuppression and a high prevalence of comorbidities (21,22). The pandemic disrupted health services, limited social interactions, and intensified existing psychosocial challenges such as loneliness, depression, and social isolation, particularly among those living alone, in residential institutions, or with limited socioeconomic resources (5,23,24).

In Croatia, the growing proportion of older people has resulted in increased demand for institutional and community-based care services. The shortage of health care workers, especially qualified nurses, is a growing challenge in the long-term care sector. However, the emigration of young people and entire families has further weakened informal support networks and intergenerational contacts, exacerbating the vulnerabilities of the older population (6,25,26).

Little is known about how marital status, place of residence and physical activity jointly shape perceived quality of life among older adults in Croatia during prolonged periods of social crisis such as the COVID-19 pandemic. Therefore, this study aimed to examine the associations between marital status, place of residence, physical activity, self-perceived health, and quality of life in older adults during the COVID-19 pandemic.

## PARTICIPANTS AND METHODS

This cross-sectional study included data from questionnaire-based surveys conducted among 962 individuals aged 65 and older during the COVID-19 pandemic, from March 11, 2020 to May 11, 2023. A total of 485 respondents were community dwelling, while 477 resided in nursing homes.

Respondents were randomly selected among community-dwelling older adults and nursing-home residents visiting family physicians at the Zagreb-Centar and Zagreb-Zapad health centers. From each health center, five family medical practices were randomly selected from the official practice lists. Within each practice, patients aged 65 years or older were selected using a systematic random sampling approach (every k-th eligible patient after a random starting point), with 50 patients included per practice, yielding a total of 500 community-dwelling respondents. Additionally, 500 participants were randomly selected from the resident lists of the affiliated nursing homes. The number of participants selected from each nursing home was determined proportionally to its size.

Individuals with physical disabilities or serious physical illnesses not directly related to the aging process (eg, malignancies, dementias such as presenile dementia, senile dementia, Alzheimer’s disease, etc) and individuals with mental illnesses were excluded. The study included only those participants who were able to care for themselves independently in performing daily activities (eg, dressing, bathing, eating, walking, etc).

Following institutional approval, participants received the questionnaires in sealed envelopes along with written instructions. Community-dwelling participants received the materials during their visits to family physicians and were instructed on how to complete and return them, given that in-clinic data collection was restricted due to epidemiological measures. In nursing homes, questionnaires were administered in designated rooms with assistance from head nurses. To improve response accuracy, participants with difficulties were interviewed face-to-face. If a selected individual was unable to participate, the next eligible person was randomly chosen.

Participants living with a partner were grouped with those who were married. Respondents were classified into four marital status categories: 1) married/living with a partner, 2) single, 3) divorced, and 4) widowed. Self-perceived health was assessed using the Short Form Health Survey-36 (SF-36), an instrument validated in the Croatian population (27). The SF-36 consists of eight dimensions: physical functioning, role limitations due to physical health, bodily pain, and general health perception, as well as role limitations due to emotional problems, social functioning, mental health, and vitality. The first four dimensions reflect physical health, while the latter represent mental health.

Personal quality of life was measured with the Personal Well-being Index (PWI) for adults, which consists of eight Likert-type scales assessing satisfaction with various life domains (28). Each domain is rated on a scale from 0 (“completely dissatisfied”) to 10 (“completely satisfied”). The PWI covers seven dimensions of subjective well-being: material well-being, health, achievement in life, personal relationships, safety (sense of security), community connectedness and sense of belonging, and future security. Physical activity levels were assessed using the short Croatian version of the International Physical Activity Questionnaire (IPAQ), which was translated and validated through a pilot study following the official protocol (29). Participants also completed a brief questionnaire on sociodemographic variables, including sex, age, accommodation, marital status, COVID-19 history, and type of treatment received.

The study was approved by the Ethics Committee of the School of Medicine, University of Zagreb. The study participants received both written and verbal information about the study and provided written informed consent before the inclusion.

### Statistical analysis

The Kolmogorov-Smirnov test indicated significant deviations from normality for all variables; however, given the large sample size, distributional properties were further evaluated using skewness and kurtosis indices. As absolute skewness values were below 3 and kurtosis values below 10, the use of parametric statistical procedures was considered appropriate.

Data are presented as means ± standard deviations. Levene’s test was used to assess the homogeneity of variances. Depending on the results, *post-hoc* pairwise comparisons were made using either the Scheffé test (when variances were equal) or the Games-Howell test (when variances were unequal). Differences in measured variables by marital status were evaluated with a one-way ANOVA or Welch’s ANOVA, as appropriate. Independent samples *t* tests were used for two-group comparisons. A two-tailed *P* value of less than 0.05 was considered statistically significant. All statistical analyses were performed with SPSS, version 8, (SPSS, Chicago, IL, USA).

## RESULTS

### Sociodemographic characteristics of participants

The study enrolled 962 older individuals (68.3% women). The most represented age group was 70-74 years (22.0%), followed by 80-84 years (20.7%), 75-79 years (18.4%), and 65-69 years (13.0%). The majority of participants were widowed (45.4%), 39.4% were married or cohabiting, 7.7% were single, and 7.4% were divorced. Overall, 50.4% of participants resided in the community and 49.6% resided in nursing homes (Table 1).

**Table 1:** Sociodemographic characteristics of respondents (N = 962)

Variable	Level	n	%
Sex	male	305	31.7
	female	657	68.3
Age (years)	65-69	125	13.0
	70-74	212	22.0
	75-79	177	18.4
	80-84	199	20.7
	85-89	143	14.9
	90-94	83	8.6
	95+	23	2.4
Marital status	married	362	37.6
	single	74	7.7
	divorced	71	7.4
	widowed	437	45.4
	living with a partner	17	1.8
	no response	1	0.1
Residence	own home	485	50.4
	nursing home	477	49.6

### Differences by marital status

Levene’s test for equality of variances showed significant differences in variances between groups for physical activity, physical functioning, social functioning, mental health, vitality, total physical health, general health perception, material well-being, achievements, interpersonal relations, and security. For these variables with unequal variances, the robust Welch test was used instead of standard ANOVA (Table 2).

**Table 2:** Equality of variances across marital status groups for domains of quality of life, health, and physical activity

Variable	Levene’s statistic	df1/df2*	p
Physical activity	311.74	3/957	<0.001
Physical functioning	3.75	3/957	0.011
Physical limitations	1.08	3/957	0.356
Emotional limitations	0.68	3/957	0.562
Social functioning	4.49	3/957	0.004
Mental health	6.96	3/957	<0.001
Vitality	4.29	3/957	0.005
Pain	2.54	3/957	0.055
Overall quality of life	1.67	3/957	0.172
Overall physical health	3.05	3/957	0.028
Overall mental health	1.83	3/957	0.141
General health perception	8.79	3/957	<0.001
Material well-being	4.19	3/957	0.006
Health	0.50	3/957	0.679
Achievements	5.26	3/957	0.001
Interpersonal relationships	10.20	3/957	<0.001
Safety	5.67	3/957	<0.001
Community conectedness	1.60	3/957	0.189
Future security	0.79	3/957	0.498

*df – degrees of freedom.

One-way ANOVA and Welch’s test revealed significant differences across marital status groups in most domains of quality of life, physical and mental health, except for perceptions of community quality and future security. Respondents who were married or cohabiting consistently reported the highest mean scores, while widowed individuals reported the lowest scores (Table 3).

**Table 3:** Differences in health, physical activity, and quality of life by marital status: one-way ANOVA and Welch’s test results*

				Married/living with a partner		Single		Divorced		Widowed	
Variable	F/W	df1/df2	p	mean	SD	mean	SD	mean	SD	mean	SD
Physical activity	198.79	3/972	<0.001	1.9^†‡§^	0.04	1.7^§^	0.09	1.5^§^	0.08	1.0	0.00
Physical functioning	33.58	3/972	<0.001	58.4^†§^	1.34	47.9	3.58	57.5^§^	3.31	40.2	1.30
Physical limitations	8.10	3/972	<0.001	49.7^§^	2.15	48.0	5.4	49.5	4.93	35.3	1.93
Emotional limitations	3.35	3/972	0.018	49.1	2.23	50.0	5.2	53.1	5.20	41.1	2.4
Social health	5.55	3/972	<0.001	55.4^§^	1.13	53.3	3.30	58.5^§^	2.89	59.5	1.21
Mental functioning	5.70	3/972	0.001	55.3^§^	0.77	52.9	2.18	52.9	2.12	51.5	0.87
Vitality	9.55	3/972	<0.001	55.0^§^	0.87	52.0	2.44	55.0^§^	2.25	48.4	0.92
Pain	3.58	3/972	0.12	51.5	1.12	54.7	2.90	55.5	2.89	48.3	1.7
General health	9.43	3/972	<0.001	52.0^§^	0.94	48.7	2.51	53.7^§^	2.15	45.5	0.90
Overall physical health	17.55	3/972	<0.001	52.9^§^	1.12	49.8	2.94	54.1^§^	2.59	42.5	1.1
Overall mental health	7.39	3/972	<0.001	59.2^§^	1.3	57.1	2.79	59.9^§^	2.51	52.7	1.2
Overall quality	5.00	3/972	0.001	8.2^‡§^	0.09	7.5	0.27	7.4	0.25	7.7	0.10
Material well-being	3.14	3/972	0.027	8.0	0.11	7.3	0.30	7.3	0.25	7.7	0.12
Health	3.17	3/972	0.024	7.0^§^	0.12	5.7	0.27	5.8	0.28	5.svi	0.12
Achievements	5.42	3/972	<0.001	7.8^‡§^	0.11	7.2	0.30	5.8	0.29	7.2	0.12
Interpersonal relations	5.48	3/972	<0.001	9.2^†‡^	0.10	8.0	0.32	8.4	0.29	9.1^†^	0.11
Safety	3.32	3/972	0.021	8.4^§^	0.11	7.9	0.30	7.8	0.31	8.0	0.12
Community	1.87	3/972	0.133	8.5	0.11	8.2	0.30	7.9	0.28	8.5	0.11
Future security	0.88	3/972	0.453	7.1	0.13	5.9	0.33	5.5	0.33	7.0	0.12

*Abbreviations: F/W – F ratio from one-way ANOVA or Welch test; df1/df2 – degrees of freedom.

†significantly higher than single (*post-hoc* analysis).

‡significantly higher than divorced (*post-hoc* analysis).

§significantly higher than widowed (*post-hoc* analysis).

Pairwise comparisons showed significant differences between the four groups of respondents categorized according to marital status on all variables, except perceived community quality of life and future security (Table 3). Participants who were married or living with a partner reported significantly higher scores than widowed participants on physical activity, physical functioning, physical limitations, social functioning, mental health, vitality, general health perception, total physical health, total mental health, overall quality of life, achievements, health, and security. Additionally, they scored significantly higher than single participants on physical functioning; higher than divorced participants on overall quality of life and achievements; and higher than all other groups on physical activity. In terms of interpersonal relationships, they also reported significantly better outcomes than single and divorced participants.

Divorced respondents demonstrated significantly better outcomes than widowed respondents on physical activity, physical functioning, social functioning, vitality, general health, total physical health perception, and total psychological health. Meanwhile, widowed respondents reported significantly better interpersonal relations than single respondents.

Although the one-way ANOVA indicated significant F-ratios for emotional limitations and material well-being, the Scheffé test did not reveal significant differences between specific groups. This may be attributed to the conservative nature of the Scheffé procedure, which often fails to detect marginal effects.

### Differences according to the place of residence

Community-dwelling respondents achieved significantly higher scores than nursing-home residents in physical activity (*t* = 10.69; *P* < 0.001), physical functioning (*t* = 10.02; *P* < 0.001), physical limitations (*t* = 3.28; *P* = 0.001), mental health (*t* = 3.03; *P* = 0.002), vitality (*t* = 3.51; *P* < 0.001), general health perception (*t* = 4.17; *P* < 0.001), total physical health (*t* = 5.82; *P* < 0.001), total mental health (*t* = 2.45; *P* = 0.014), achievements (*t* = 2.48; *P* = 0.013). For the variable “security in the future,” the opposite was observed (*t* = −2.19; *P* = 0.029) (Table 4).

**Table 4:** Differences in health, physical activity, and quality of life between community-dwelling respondents and nursing-home residents

			Residing in one’s own home		Residing in nursing home	
Variable	t	p	mean	SD	mean	SD
Physical activity	10.69	<0.001	1.7	0.77	1.2	0.56
Physical functioning	10.02	<0.001	57.9	26.70	40.4	27.46
Physical limitations	3.21	0.001	47.7	41.97	39.2	41.01
Emotional limitations	1.24	0.214	47.5	42.76	44.0	43.64
Social functioning	1.52	0.128	64.4	23.65	62.0	25.24
Mental health	3.03	0.002	65.3	15.93	62.0	18.11
Vitality	3.51	<0.001	53.8	18.04	49.6	19.18
Pain	1.34	0.182	51.5	21.71	49.6	23.44
General health	4.17	<0.001	51.5	18.23	46.4	19.53
Overall physical health	5.82	<0.001	52.2	22.09	43.9	21.99
Overall mental health	2.45	0.014	57.8	20.83	54.4	21.59
Overall quality	1.34	0.180	7.9	1.83	7.8	2.24
Material well-being	1.13	0.259	7.8	2.17	7.6	2.51
Health	1.05	0.293	6.8	2.26	6.6	2.49
Achievements	2.48	0.013	7.6	2.20	7.2	2.63
Interpersonal relations	1.22	0.222	9.1	1.95	8.9	2.54
Safety	−0.12	0.901	8.1	2.23	8.2	2.50
Community connectedness	−1.32	0.189	8.3	2.23	8.5	2.35
Future security	−2.19	0.029	6.8	2.55	7.2	2.61

## DISCUSSION

This study highlights a significant influence of marital status and living arrangements on various domains of quality of life, health, and well-being in older adults. Specifically, married and community-dwelling individuals exhibited superior physical functioning, higher vitality, and greater overall life satisfaction than their widowed or institutionalized peers. These findings underscore the protective role of intimate social bonds, companionship, and independent living, which promote resilience and psychological well-being in late adulthood.

Marital status has been recognized as a significant predictor of self-perceived physical health (30). Married or cohabiting individuals consistently demonstrate higher life satisfaction, enhanced physical and mental well-being, and increased engagement in health-promoting behaviors compared with their single, divorced, or widowed counterparts (31,32). Marriage provides emotional support, companionship, and opportunities for engagement in cognitively stimulating activities, all of which contribute to enhanced mental and cognitive well-being (33). However, beyond the categorical classification of marital status, it is essential to consider the quality and stability of the relationship, as well as the individual’s subjective satisfaction (34). In line with these findings, our data reveal that older adults who were married or cohabiting scored significantly higher on social functioning, emotional vitality, and overall mental health compared with widowed individuals. Similarly, divorced participants also reported better outcomes than the widowed group in terms of social functioning, vitality, and overall mental health. Widowed individuals more frequently negatively perceive their physical health, and often state that their health limitations interfere with daily functioning. These findings can be attributed to the emotional burden associated with spousal loss, a life event known to compromise psychological resilience. Widowed and never-married individuals exhibit reduced functional capacity and lower engagement in health-promoting activities (35).

Moreover, robust social ties, particularly sustained familial contact, are strongly associated with increased longevity, preserved cognitive function, and a slower rate of health decline in later life. In contrast, social isolation is a well-documented risk factor for both psychological distress and functional deterioration (36). Sustaining meaningful social relationships not only improves quality of life in late adulthood but also contributes to extended lifespan and preserved health. Socially active older adults are more likely to maintain cognitive performance and independence (37).

While many older individuals exhibit psychological resilience and adaptive coping following spousal loss, a substantial proportion face heightened risks of mental health deterioration, decreased functional capacity, and even premature mortality risks further exacerbated by pandemic-related restrictions. The COVID-19 pandemic has further compounded this vulnerability, as prolonged grief and social isolation have emerged as risk factors for cognitive decline in older adults (38).

In elderly individuals, family relationships often become the most enduring and reliable form of social connection. Friendships also serve as a source of well-being; however, some studies suggest that older adults often fear becoming a burden to their loved ones and friends, which may limit their participation in social interactions (39,40).

Few studies have examined the interplay of marital status and living arrangements in the quality of life of older adults, specifically during the COVID-19 pandemic. Emotional stress, intensified by the COVID-19 pandemic, represented an additional risk factor for diminished well-being in late life. Anxiety has been shown to adversely affect individuals aged 75 and older. In addition to the psychological toll of isolation, the reduced ability to engage in physical activity during lockdown further compounded health risks, not only among nursing home residents but also among community-dwelling older adults (41). In our study, community-dwelling older adults reported significantly better outcomes on physical activity, physical functioning, mental health, and perceptions of general health and future well-being, which highlights the value of autonomy and environmental familiarity in supporting holistic health. Interestingly, the only domain in which nursing home residents reported higher scores was future security. This suggests that despite worse physical and mental health, respondents may perceive a greater sense of safety or care continuity within the institutional environment. The literature consistently shows that older community-dwelling adults report better general health, greater vitality and energy, as well as enhanced social functioning than nursing-home residents (42). Social engagement and autonomy may help community-dwelling individuals maintain daily functioning and preserve quality of life.

The COVID-19 pandemic posed significant challenges for long-term care facilities. Due to the communal nature of these environments, nursing homes experienced rapid viral transmission, high mortality rates, and disruptions in routine care. Strict isolation protocols, including visiting bans and limited access to digital communication tools, reduced residents’ social interaction and emotional support systems (43,44). These measures, although necessary for infection control, deprived residents of autonomy, connectedness, and participation in meaningful activities (45). In contrast, older adults living in private households, particularly those who live with a spouse or family members, were better equipped to withstand the psychosocial effects of the pandemic. Such living arrangements may serve as a protective factor, buffering against loneliness and supporting overall quality of life during crises (46). Our findings are consistent with this observation.

Furthermore, moderate physical activity, especially when it involves cognitive and social engagement, significantly contributes to the preservation of functional ability required for independent living (47). Social isolation during the pandemic negatively affected quality of life. However, in our study, participants with higher levels of physical activity and better health maintained a superior quality of life even during prolonged isolation. These results underscore the potentially severe consequences of even short periods of social isolation on the mental health and emotional well-being of older adults (46).

The COVID-19 pandemic has highlighted the particular vulnerability of older adults, especially those in long-term care facilities, and the need for policies that strengthen social connectedness and autonomy in later life. Community-based interventions should support social participation, physical activity and a sense of safety among older people, while longitudinal research is needed to clarify the long-term effects of marital disruption and institutionalization on health and quality of life. Together, these findings support aging-in-place strategies and psychosocial support systems that protect the dignity, independence, and well-being of older adults during public health emergencies and beyond.

Several limitations of the study should be acknowledged. Older participants frequently experienced difficulty maintaining attention throughout the survey, which necessitated the adaptation of the questionnaire by using shorter and simpler questions to enhance comprehension and ensure completion. Additionally, due to participants’ reluctance to disclose personal information, key objective variables such as income level, the presence of chronic illnesses, and the history of hospitalizations were excluded from the questionnaire. The absence of these data may have limited the depth of the analysis and reduced the overall comprehensiveness and generalizability of the study’s findings. Another limitation is the lack of data on the participants' functional independence and some other key gerontological indicators. Furthermore, the analysis relied on self-report measures of health status and physical activity. While more objective assessment methods exist, self-report questionnaires remain the most widely used instruments in population-based and epidemiological research due to their practicality, cost-effectiveness, and ability to capture subjective health perceptions. Moreover, when evaluating individuals’ personal experiences, well-being, and life satisfaction, self-assessment represents a valid and appropriate methodological approach. Also, the SF-36 instrument has no known limitations when applied to nursing-home residents, and the IPAQ questionnaire is well-validated and suitable for use in older populations.

In conclusion, marital status and living arrangements significantly affected the self-perceived health, physical activity levels, and quality of life of older adults during the COVID-19 pandemic. Understanding the complex interplay between marital status, residential environment, and physical activity in shaping perceived quality of life among older adults, particularly during periods of social disruption such as the COVID-19 pandemic, is crucial for designing targeted interventions and evidence-based public health policies. Such insights can guide efforts to promote healthy aging, enhance resilience, and support the psychological and social well-being of the aging population in future public health crises.
